# A Chromosome-Level Genome Assembly of the Non-Hematophagous Leech *Whitmania pigra* (Whitman 1884): Identification and Expression Analysis of Antithrombotic Genes

**DOI:** 10.3390/genes15020164

**Published:** 2024-01-26

**Authors:** Zichao Liu, Fang Zhao, Zuhao Huang, Bo He, Kaiqing Liu, Feng Shi, Zheng Zhao, Gonghua Lin

**Affiliations:** 1 Engineering Research Center for Exploitation and Utilization of Leech Resources in Universities of Yunnan Province, School of Agriculture and Life Sciences, Kunming University, Kunming 650214, China; abclzc@aliyun.com (Z.L.); lkqkm@163.com (K.L.); sf14787788954@163.com (F.S.); 2 School of Life Sciences, Jinggangshan University, Ji’an 343009, China; zf_lgh@163.com (F.Z.); hzhow@163.com (Z.H.); hebo90@126.com (B.H.); 3 Key Laboratory of River and Lake Ecological Health Assessment and Restoration in Yunnan Province, Kunming Dianchi Lake Environmental Protection Collaborative Research Center, Kunming University, Kunming 650214, China; zhaozheng2118@163.com

**Keywords:** non-hematophagous leech, antithrombotic protein, antithrombotic gene, sequence similarity, RNA-seq, gene expression

## Abstract

Despite being a non-hematophagous leech, *Whitmania pigra* is widely used in traditional Chinese medicine for the treatment of antithrombotic diseases. In this study, we provide a high quality genome of *W. pigra* and based on which, we performed a systematic identification of the potential antithrombotic genes and their corresponding proteins. We identified twenty antithrombotic gene families including thirteen coagulation inhibitors, three platelet aggregation inhibitors, three fibrinolysis enhancers, and one tissue penetration enhancer. Unexpectedly, a total of 79 antithrombotic genes were identified, more than a typical blood-feeding *Hirudinaria manillensis*, which had only 72 antithrombotic genes. In addition, combining with the RNA-seq data of *W. pigra* and *H. manillensis*, we calculated the expression levels of antithrombotic genes of the two species. Five and four gene families had significantly higher and lower expression levels in *W. pigra* than in *H. manillensis*, respectively. These results showed that the number and expression level of antithrombotic genes of a non-hematophagous leech are not always less than those of a hematophagous leech. Our study provides the most comprehensive collection of antithrombotic biomacromolecules from a non-hematophagous leech to date and will significantly enhance the investigation and utilization of leech derivatives in thrombosis therapy research and pharmaceutical applications.

## 1. Introduction

Thrombosis, the formation of a blood clot within a blood vessel, is a serious medical condition that can lead to life-threatening complications. It occurs when blood platelets and fibrin accumulate at the site of an injury or abnormal vessel wall, obstructing the flow of blood and causing ischemic and hypoxic tissue damage [1]. There are two main types of thrombosis: arterial thrombosis, which affects blood vessels carrying oxygenated blood to tissues, and venous thrombosis, which occurs in blood vessels returning deoxygenated blood to the heart. Thrombosis can lead to serious complications, such as heart attacks, strokes, and deep vein thrombosis, which cause over 15 million deaths per year worldwide [2]. Treatment for thrombosis varies depending on the severity and location of the clot, as well as the patient’s overall health.

Antithrombotic drugs, including anticoagulant, antiplatelet, and fibrinolytic medications, are commonly used to prevent and treat thrombosis. These medications help reduce the risk of clot formation by inhibiting platelet function or interfering with the blood clotting process [3]. Although antithrombotic medications have mitigated thrombotic incidents among patients, their capacity to minimize thrombotic, disease-related fatalities is limited. This predominantly stems from their dependence on a single-action target drug, making it challenging to accommodate dosage disparities between individuals. Consequently, instances of drug resistance, internal bleeding, and liver or kidney damage become common, amongst other severe adverse effects that pose risks to patients’ lives. Anticoagulant drugs such as warfarin [4], antiplatelet aggregation drugs such as clopidogrel [5], and fibrinolytic medications like alteplase [6] were repeatedly reported to have caused various side effects. Thus, it is crucial to conduct extensive research and development to establish safe and efficient multi-target medications with minimal adverse effects for the therapy of thrombotic disorders.

Leeches, belonging to the class of annelid worms, are unique and fascinating creatures known for their medicinal and ecological significance. These small, segmented worms possess a sucker at each end of their bodies, which enables them to attach to host organisms and suck their blood. This unusual feeding habit has made leeches an essential tool in various scientific fields, particularly in medicine, biology, and ecology [7,8]. In the medical realm, leeches have been used for centuries to treat a range of conditions, including inflammation, pain, and blood disorders. Their saliva contains a cocktail of powerful compounds that promote blood flow, reduce inflammation, and prevent clotting [9]. These bioactive substances could have significant medical and medicinal value if utilized effectively. In traditional Chinese medicine, a few species of leeches known as “Shuizhi” are frequently used to treat and prevent thrombotic illnesses [10].

While the primary aspect of leech behavior is the sanguivory of specific species, the remaining species also engage in other feeding habits, such as macrophagy and omnivory [11]. For example, although the highly specialized sanguivory life-history mode is prevalent, it is not held constant and can be lost multiple times during the evolutionary history of leeches [12]. The ancestral Hirudinidae is believed to have been a blood feeder, while at least two genera, *Whitmania* and *Haemopis*, have transitioned to become invertebrate predators [11,13]. Of particular interest are the species in the *Whitmania* genus, including *W. pigra*, *Whitmania acranulata*, and *Whitmania laevis*, which are sympatrically distributed in Chinese drainages. *W. pigra* preys exclusively on snails, *W. acranulata* consumes both aquatic earthworms and insect larvae, and *W. laevis* has a broader diet that comprises snails and insect larvae, according to Yang’s 1996 study [14].

The Pharmacopeia of the People’s Republic of China (PPRC) is a crucial element in China’s drug laws and regulations, and materials not listed in the PPRC should not be used for medicinal purposes in theory [15]. The present edition of the PPRC designates three leech species (*Hirudo nipponia*, *W. pigra*, and *W. acranulata*) as the legal materials for “Shuizhi” products. Among the three species, *W. pigra*, with the largest body size and most plentiful resources, has become the primary material for “Shuizhi”. It is worth noting that there has been a long-standing debate about whether *W. pigra* can serve as the fundamental source of “Shuizhi” due to its non-bloodsucking habits [16]. Some researchers contend that the antithrombotic capabilities of *W. pigra* were likely lost during its shift from sanguivory to macrophagy, and thus this species should be excluded from the PPRC [17]. Conversely, other researchers maintain that *W. pigra* should be included in the PPRC due to its anticoagulant properties, albeit weaker than those of sanguivorous leeches [18], and antiplatelet aggregation abilities [19,20]. Recent studies support that at least one type of hirudin from the *W. pigra* exhibited anticoagulant activity [21].

With the advancement of high-throughput sequencing technology, the study of leech genes has entered the era of genomics. The genomes of several leech species, such as *Helobdella robusta* [22], *H. medicinalis* [23,24], *Hirudinaria manillensis* [25,26], have been published. Recently, we used state-of-the-art third-generation sequencing (PacBio HiFi) and next-generation sequencing (Illumina Hi-C, Survey and RNA-seq) to obtain a nearly complete chromosome-scale genome of a blood-feeding *H. manillensis*. Based on the high-quality genome, we systematically identified 72 antithrombotic genes involving 21 gene families [27], many of which were not predicted in previous studies.

In total, three genomes of *W. pigra* have been sequenced. Tong et al. [28] utilized next-generation sequencing methods to produce a draft assembly (GenBank accession: GCA_021650995.1) of 177 Mb, consisting of 10,050 scaffolds with an N50 of 728 kp. Using the Nanopore PromethION platform, Zheng et al. [26] recently published another genome of the *W. pigra*. Using Hi-C technology, the authors obtained a chromosome-level genome measuring 181.4 Mb in total length with 194 scaffolds and a significantly larger N50 value of 16.2 Mb. The authors focused primarily on gene expression patterns before and after bloodsucking and did not conduct a systematic analysis of antithrombotic genes. An additional genome of the *W. pigra* was deposited by a research group from the Chinese Academy of Medical Sciences and Peking Union Medical College (GCA_021613335.1), but regrettably, no further analyses were obtainable for this data. It should be noted that, probably due to the low quality of the genome assembly and the structural complexity of antithrombotic genes, certain genes were likely omitted during process-oriented genome annotation. Using the well-known anticoagulant hirudin as an example, Tong et al. [28] and Zheng et al. [26] identified only two and one hirudin coding genes in their genomes, respectively. However, our investigation in the present study revealed at least seven hirudin coding genes in the *W. pigra* genome (see below).

Here, we used the third generation (PacBio HiFi) and the next-generation sequencing methods (Illumina Hi-C, Survey and RNA-Seq) to obtain a nearly complete chromosome-scale genome of the *W. pigra*. With a so called BRAKER-plus gene prediction strategy which combined process-oriented and manual prediction approaches [27], we systematically identified the antithrombotic related genes of *W. pigra*. Combined with the results from *H. manillensis* [27], we compared the gene constitutions between the non-hematophagous and hematophagous leeches. Meanwhile, we provided RNA-seq data of the *W. pigra* and *H. manillensis* and calculated the expression levels of the antithrombotic genes of the two species. We aim to show the similarities and differences in the expression characteristics of the antithrombotic genes between the non-hematophagous and the hematophagous leeches.

## 2. Materials and Methods

### 2.1. DNA and RNA Sequencing

*W. pigra* individuals were live trapped from Yutai County, Shandong Province, China (GPS Coordinates: E 116°39′17″, N 34°57′47″). After removing the digestive tracts, total genomic DNA was isolated from fresh tissues using the DNeasy Blood and Tissue Kit (Qiagen, Chatsworth, CA, USA). The collected DNA was assessed for quality and integrity through agarose gel electrophoresis, NanoDrop spectrophotometry (NanoDrop Technologies, Wilmington, DE, USA), and Qubit fluorometry (Thermo Fisher Scientific, Waltham, MA, USA). Once the DNA met the required standards of quality and quantity, it was used to construct the PacBio and Illumina libraries.

The DNA and RNA sequencing were performed with reference to our recently published article [27]. Briefly, a HiFi SMRTbell library was created using the SMRTbell Express Template Prep Kit 2.0 (PacBio, Menlo Park, CA, USA) and were sequenced by the PacBio Sequel II platform. HiFi reads were generated using the CCS software (https://github.com/PacificBiosciences/ccs, accessed on 19 April 2022) with default settings. A Hi-C library was created using HindIII restriction endonuclease. Paired-end sequencing of the Hi-C reads was performed using the Illumina HiSeq 2000 sequencing platform (Illumina Inc., San Diego, CA, USA) with both directions of 150 bp reads. We also generated short Illumina reads (called survey reads) for polishing the genome assembly. A DNA library with ~350 bp insertions were constructed and were then sequenced with both directions of 150 bp reads. For RNA-seq, total RNA was extracted from head tissue using the QIAGEN RNeasy Plant Mini Kit (QIAGEN, Hilden, Germany). The cDNA library was prepared using the TruSeq Sample Preparation Kit (Illumina, San Diego, CA, USA), and paired-end sequencing with 150 bp was conducted on the Illumina HiSeq 2000 sequencing platform.

### 2.2. Genome Assembling

The genome was assembled using HiFi reads by NextDenovo V2.5.0 [29] and was polished using survey reads by NextPolish v1.4.0 [30]. Hi-C reads were used to generate a chromosome-level assembly using the YaHS v1.1a program [31]. The resulting YaHS-generated files were then imported into Juicebox v1.11.08 [32] for visualizing the Hi-C maps and manual fine-tuning. The final chromosome-level assembly was finally created using Juicer v1.6.2 [33]. Furthermore, we utilized survey reads for mitochondrial genome assembly through GetOrganelle v1.7.7.0 [34].

We employed BUSCO v.4.1.4 [35] and Merqury [36] to assess the thoroughness and quality of the genome assembly, respectively. We utilized both a de novo and homology approach to identify repetitive sequences in the genome. A de novo library was first constructed using RepeatModeler v2.0.3 [37], which was later merged with the Annelida repeat sequences extracted from the RepBase database v20181026 [38]. RepeatMasker v4.1.2-pl [37] was then engaged to search for repeat sequences from the genome, and the repeat-masked genomes were further utilized for the prediction of protein-coding genes.

### 2.3. Gene Prediction

According to our recent study, a so-called BRAKER-plus strategy [27], which combined BRAKER prediction [39] and our manual prediction, was used for identifying antithrombotic genes. STAR v2.7.9a [40] was utilized to map the RNA-seq reads to the repeat-masked genome, and the protein-coding genes were predicted using BRAKER v2.1.6 with default settings [39]. Meanwhile, the RNA-seq data of the *W. pigra* sequenced in this study and those from GenBank (SRR15881153~SRR15881157 and SRR15881159~SRR15881165) were assembled using Trinity v2.9.0 [41] and the protein-coding genes were predicted using GeneMarkS-T v5.1 [42]. All available antithrombotic genes and/or proteins from the published literatures were collected and used as queries for blasting the BRAKER-derived and the RNA-seq-derived coding sequences (CDS). After duplication removal, manually predicted antithrombotic CDS were mapped to the repeat-masked genome using Exonerate v2.2.0 with the est2genome model [43]. The GFF files from the process oriented BRAKER prediction and the manual prediction were merged using AGAT v1.2.0 [44]. After manually cleaning the duplicated features, we obtained a final version of the GFF file (BRAKER-plus.gff), which had updated coordinate information on all of the potential antithrombotic genes.

The antithrombotic proteins were extracted using GffRead v0.12.7 [45]. The potential signal peptide region was predicted using SinalP v6 [46]. Each protein family and its corresponding archetypal proteins [27] were consolidated, and after aligning using MEGA v11.0.13 [47], the pairwise longest similarity index values were calculated using EMBOSS v 6.6.0.0 [48] To elucidate the relationships among the members of large protein families, we also reconstructed phylogenetic trees using IQ-TREE v1.6.12 with default settings [49].

### 2.4. Expression of Antithrombotic Genes

Using the RNA-seq data sequenced in this study and those from previous studies, we compared expression characteristics of antithrombotic genes between the non-hematophagous (*W. pigra*) and hematophagous (*H. manillensis*) leeches. Besides the RNA-seq data used for gene prediction (SRR26513850 and SRR26151944, respectively, for *W. pigra* and *H. manillensis*), three additional samples from the leech head were sequenced for *W. pigra* (SRR26541743~SRR26541745) and *H. manillensis* (SRR26541746, SRR26541752, and SRR26541752) each. Moreover, RNA-seq data of the oral sucker of three adult *W. pigra* (SRR15881156, SRR15881157, and SRR15881159) and *H. manillensis* (SRR15881208~SRR15881210) each were also used. As a result, RNA-seq data from seven *W. pigra* and seven *H. manillensis* samples were used for gene expression analysis.

The CDS of all predicted genes (including the antithrombotic genes) of the *W. pigra* were used as references. The salmon program v1.0.0 [50] was used to calculate the transcripts per million value (TPM) for every gene, based on a RNA-seq of each *W. pigra* sample. The same methods were applied to each *H. manillensis* sample. In order to increase comparability, the TPM of the genes from the same gene family were grouped. It should be noted that the members in the *hirustasin* superfamily were too closely related to be clear distinguished; thus, we combined TPM of all the members into a single group.

In order to compare the total gene expression level of each antithrombotic gene family between the two leech species for each RNA-seq sample, we summed the TPM of all members in each family. The summed total TPM of each gene family (named as tTPM) were then compared between the *W. pigra* and *H. manillensis* samples using the non-parametric Mann–Whitney U test in SPSS v25.0 (IBM Corp., Armonk, NY, USA). Moreover, based on the tTPM values of each sample, we used the hierarchical cluster analysis in SPSS to compare the expression spectra of the antithrombotic gene families between the *W. pigra* and *H. manillensis*.

## 3. Results

### 3.1. Basic Information of Genome Assembly

**Figure 1:**
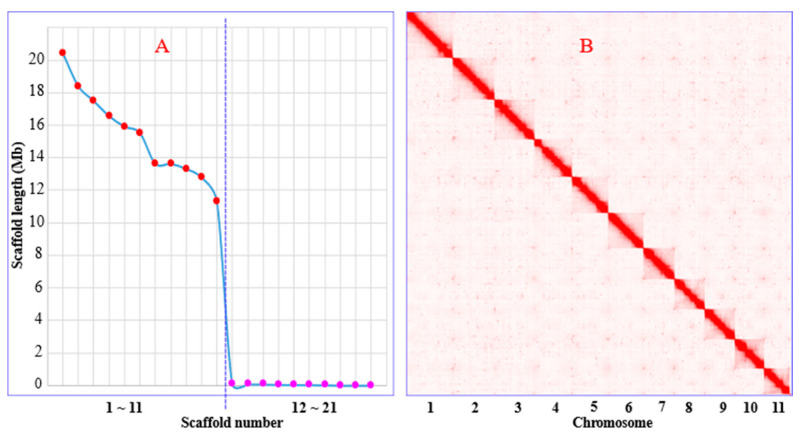
Assembly information of the *W. pigra* genome. (**A**) Scaffold length distribution (the red dots indicate the 11 long scaffolds; the pink dots indicate the remaining short scaffolds); (**B**) Hi-C links among pseudo-chromosomes (darker red color indicates higher contact probability).

We obtained a total of 35.65 Gb of high-precision HiFi reads with an average length of 11.58 Kb. The de novo assembly yielded 62 contigs, summing up to a length of 173.56 Mb (N50 = 9.88 Mb). Additionally, 20.93 Gb of Hi-C reads were sequenced, allowing for the anchoring of the contigs into 21 scaffolds, measuring a total length of 169.35 Mb (N50 = 15.91 Mb). The first 11 longest scaffolds ranged from 20.41 to 11.30 Mb, while the remaining 10 debris were each below 0.1 Mb. Referring to the well-resolved Hi-C maps and severely discontinuous length distribution between the long scaffolds and the short debris (Figure 1), we inferred the 11 long scaffolds as pseudo-chromosomes, which constitutes 99.77% of the total scaffold length. We also sequenced 18.99 Gb of the NGS reads and assembled a circular complete mitochondrial genome of 15,985 bp. As a result, we obtained a nearly complete genome of the *W. pigra*, totaling ~170 Mb in length, comprising eleven pseudo-chromosomes, one mitochondrial genome, and nine debris (0.39 Mb). The final genome assembly was available as Supplementary Material File S1.

A BUSCO analysis showed that of the 255 BUSCOs, 250 (98.04%) were captured, including 235 (92.16%) complete and single-copy BUSCOs, 10 (3.92%) complete and duplicated BUSCOs, and 5 (1.96%) fragmented BUSCOs, while only 5 (1.96%) BUSCOs were missed. A Merqury assessment showed that the quality score of our genome was 43.47. Repeat sequence analyses using the RepeatModeler and RepeatMasker showed that a total of 27.02% sites were identified as repeats, including 7.82% retroelements, 6.80% DNA transposons, 0.24% rolling circles, and 9.46% unclassified repeat elements.

### 3.2. Antithrombotic Genes and Proteins

Based on the BRAKER-plus strategy, 24,156 protein-coding genes were predicted, with total length of 35,058,818 bp, and with a N50 length of 2076. A total of 20 gene families consisting of 79 genes were identified (Table 1). The GFF file (File S2) and all predicted CDS (File S3), and the CDS of the 79 antithrombotic genes (File S4), were available as Supplementary Material files.

In total, 14 of the 20 corresponding protein families were coagulation inhibitor, which could be further categorized into three groups: (1) thrombin inhibitors including hirudin and progranulin; (2) Factor Xa inhibitors including antistasin, lefaxin, and therostasin; (3) and serine protease inhibitors including hirustasin, hirustasin-like, guamerin, piguamerin, bdellastasin, eglin, bdellin, leech-derived tryptase inhibitor (LDTI), and *Hirudinaria manillensis* elastase inhibitor (HMEI). The three families were platelet aggregation inhibitor, including saratin, apyrase, and lumbrokinase. The three families were fibrinolysis enhancers, including destabilase, γ-glutamyl transpeptidase (GGT), and leech carboxypeptidase inhibitor (LCI). The remaining family, hyaluronidase, was a tissue penetration enhancer (Table 1). For more information on the functions of these protein families, also see the previous paper [27] and the references, therein. All proteins had a sequence similarity of over 50% with their corresponding archetypal proteins (see the alignments below).

**Figure 2:**
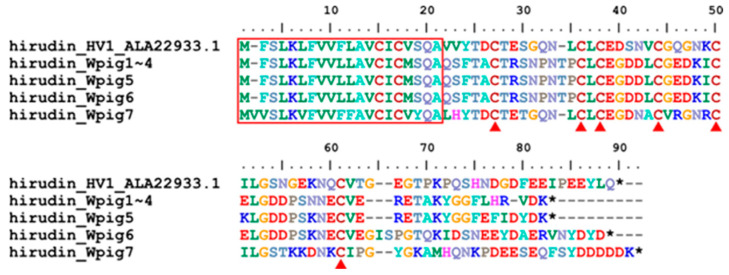
Sequence alignment of hirudins. The archetypal hirudin was from *Hirudo medicinalis*, while the seven hirudins were identified from the *W. pigra* genome. The four hirudins, hirudin_Wpig1~hirudin_Wpig4, were identical. The red frame indicates a signal peptide region; the red triangles show conserved cysteine residues; the black stars mean stop codons.

Unlike *H. manillensis*, which had five hirudin genes [27], seven *hirudins* (*hirudin_Wpig1*~*hirudin_Wpig7*) were identified from the *W. pigra* genome. A total of four genes (*hirudin_Wpig1*~*hirudin_Wpig4*) were identical in both the DNA and protein sequences (Figure 2). Online blasting in GenBank showed that six of the corresponding hirudins were identical to the reported hirudins, while the remaining (hirudin_Wpig5) were nearly identical to the reported hirudin (Table 2).

**Figure 3:**
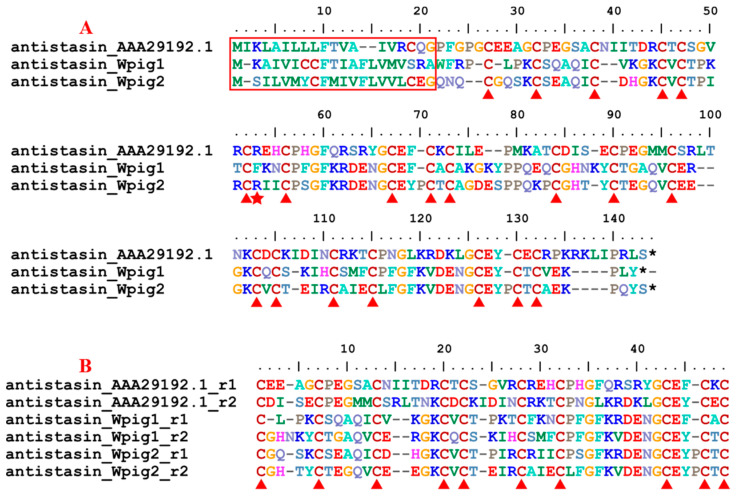
Sequence alignment of antistasins. (**A**) Alignment of the antistasin first discovered in *Haementeria officinalis* and the antistasins identified from the *W. pigra* genome; (**B**) Alignment of the internal tandem repeats of the two *W. pigra* antistasins. The red frame indicates a signal peptide region; the red triangles show conserved cysteine residues; the black stars mean stop codons; the red star shows the catalytic residue arginine.

Same as the *H. manillensis*, one progranulin gene, two antistasin gene, three lefaxin genes, and one therostasin gene were detected in the *W. pigra* genome. A signal peptide region was present in the protein families progranulin, antistasin and therostasin, but not in the remaining lefaxin. Internal tandem repeats were found in the progranulin and antistasin families. The catalytic residue arginine [51] was conserved in antistasin_Wpig2 but not in antistasin_Wpig1. In contrast, in *H. manillensis*, the catalytic arginine of both antistasins were conserved [27]. For each protein family, the alignment of the protein(s) and the corresponding archetypal protein was shown in Figure 3 and Figures S1–S3.

**Figure 4:**
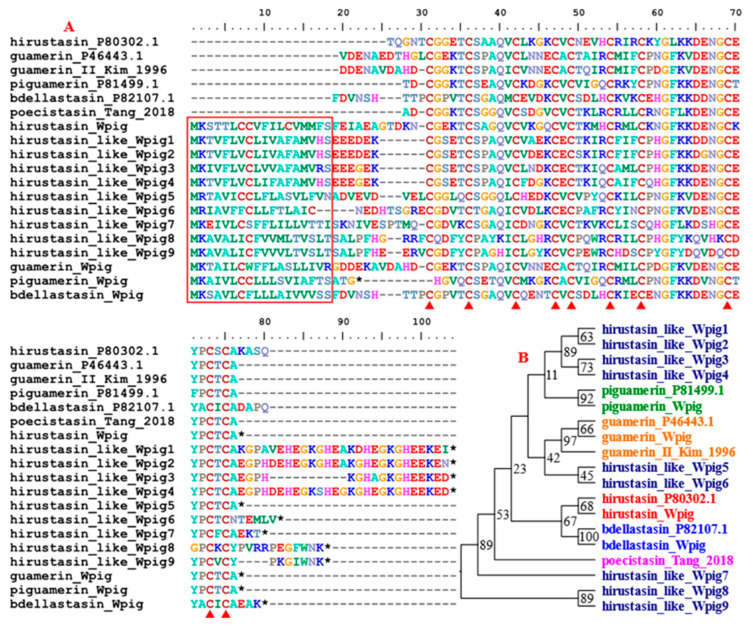
Sequence alignment and phylogenetic relationship of the hirustasin super-family. (**A**) Alignment of the archetypal hirustasin (*H. medicinalis*), guamerin (*H. nipponia*) and piguamerin (*H. nipponia*), bdellastasin (*H. medicinalis*), poecistasin (*H. manillensis*) and their homologues from the *W. pigra* genome. The red frame indicates a signal peptide region; the red triangles show conserved cysteine residues; the black stars mean stop codons. (**B**) Phylogenetic relationship among the members in the hirustasin super family. The numbers beside each node in the tree represent bootstrap percentages calculated by maximum likelihood analysis.

Our recent study indicated that the gene families *hirustasin*, *hirustasin-like*, *guamerin*, *piguamerin*, *bdellastasin*, and *poecistasin* were closely related and can be grouped into hirustasin superfamily [27]. A total of 13 genes from the superfamily were detected in the *W. pigra* genome, less than those from *H. manillensis* which had 18 genes. We combined all of the proteins identified in this study and their corresponding archetypal proteins to determine their affiliation by phylogenetic analysis. The results showed that one hirustasin, one guamerin, one piguamerin, and one bdellastasin, but no poecistasin, were found (Figure 4). The remaining nine members did not cluster with any of the archetypal proteins, we temporally named them as hirustasin-like proteins. All proteins had a signal peptide and 10 conserved cysteines.

Two eglin genes, one bdellin gene, and one LDTI gene were identified from the *W. pigra* genome. All of the three corresponding protein families (Figures S4–S6) included a signal peptide region. The bdellin and LDTI had six and twelve conserved cysteines, but the eglins had no conserved cysteine site. The bdellin had a C-terminal region with a highly charged repetitive sequence (Figure S5). Similar to the antistasin mentioned above, the LDTI also contains a twofold internal repeat with six cysteines.

A total of 20 HMEIs genes were detected in the *W. pigra* genome. (Figure S7), similar to *H. manillensis* (*N* = 18), confirming that that they are members of a large gene family. Although the corresponding proteins of these genes were highly variable, they all had significant common features including a signal peptide and about ten conserved cysteines. At the protein sequence level, HMEI_Wpig05 and HMEI_Wpig06 were identical, while HMEI_Wpig12 and HMEI_Wpig13 were identical. The gene *HMEI_Wpig07* had a deletion of one base, resulting in a frameshift mutation and an early termination in the protein sequence.

**Figure 5:**
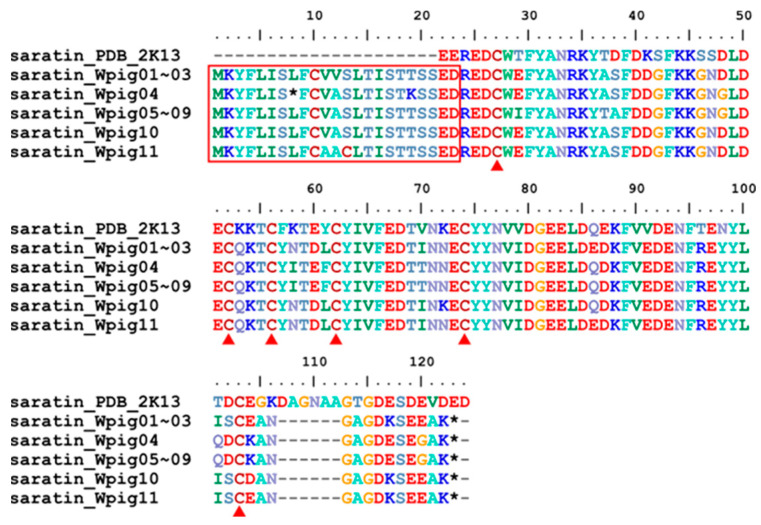
Sequence alignment of the saratins. Alignment of the archetypal saratin discovered from *H. officinalis* and the 11 saratins identified from the *W. pigra* genome. The three proteins saratin_Wpig01~saratin_Wpig03 were identical; the five proteins saratin_Wpig05~saratin_Wpig09 were identical. The red frame indicates a signal peptide region; the red triangles show conserved cysteine residues; the black stars mean stop codons.

A total of eleven saratin genes were detected in the *W. pigra* genome, far beyond the number (*N* = 2) of those from the *H. manillensis*. Only one gene had been pseudogenetic with an in frame stop codon in the signal peptide region (Figure 5). Overall, three apyrase genes were identified from *W. pigra*, less than those from *H. manillensis* (*N* = 5). In contrast, four lumbrokinase genes were detected in the *W. pigra*, more than those from *H. manillensis* (*N* = 3). The corresponding proteins saratins (Figure 5), apyrases (Figure S8) and lumbrokinases (Figure S9) had six, two, and eight conserved cysteines, respectively. All of these proteins had a signal peptide region.

**Figure 6:**
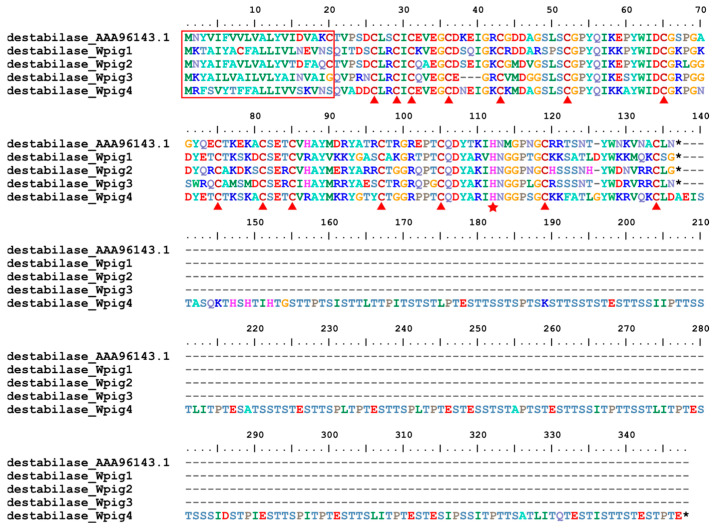
Sequence alignment of destabilases. Alignment of the archetypal destabilase from *H. medicinalis* and those found in the *W. pigra* genome. The red frame indicates a signal peptide region; the red triangles show conserved cysteine residues; the black stars mean stop codons; the red star shows the catalytic residue.

In total, four destabilase genes were detected in the *W. pigra* genome, more than those from *H. manillensis* (*N* = 3). Similar to those from *H. manillensis*, the N-terminal of the corresponding protein were relatively conserved; however, the C-terminal of one protein (destabilase_Wpig4) was largely elongated. A total of two GGT genes were found in the *W. pigra* genome, also more than those from *H. manillensis* (*N* = 2). However, the *GGT_Wpig2* was a pseudogene, with one early termination codon. Same as *H. manillensis*, a single LCI gene was detected in the *W. pigra* genomes. The corresponding protein families destabilase (Figure 6), GGT (Figure S10), and LCI (Figure S11) had fourteen, six, and eight conserved cysteines. The destabilases and GGTs, but not the LCI, had a signal peptide. Moreover, the catalytic residues, histidine of destabilases [52], and threonine of GGTs [53] from both the *W. pigra* and *H. manillensis* were conserved.

**Figure 7:**
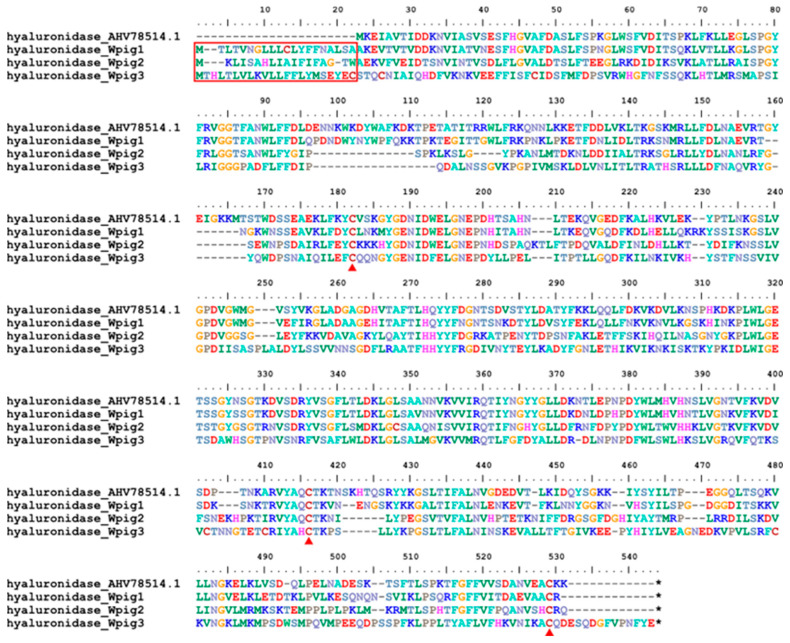
Sequence alignment of hyaluronidases. Alignment of the hyaluronidase first discovered from *H. nipponia* and those identified from the *W. pigra* genome. The red frame indicates a signal peptide region; the red triangles show conserved cysteine residues; the black stars mean stop codons.

Like *H. manillensis*, three hyaluronidase genes were identified in *W. pigra*. A signal peptide and two conserved cysteines were detected in the corresponding proteins (Figure 7).

### 3.3. Gene Expression

We used RNA-seq data to estimate the relative expression levels (TPM) of the antithrombotic genes and the other protein-coding genes predicted from the genomes (Table 3). For *H. manillensis*, the average TPM of the 72 antithrombotic genes was 680.3 ± 134.2, about 18 times of the average TPM of the other protein-coding genes (37.6 ± 0.4); while for *W. pigra*, the average TPM of the 79 antithrombotic genes was 1099.8 ± 562.2, about 29 times the average TPM of the other protein-coding genes (37.9 ± 1.8). The Mann–Whitney U test showed that the average TPM of the antithrombotic genes of *W. pigra* was significantly larger than that of *H. manillensis* (*Z* = −2.364, *p* = 0.017); but no significant deviations were found on the average TPM of the other protein-coding genes (*Z* = −1.345, *p* = 0.209). 

In order to show the overall expression level of each antithrombotic gene family for each sample, we summed the TPM of all members in each family (tTPM). The mean ± SD values of the tTPM of each species were listed in Table 4. For the *H. manillensis* samples, the *lefaxin* gene family had the highest expression level (18,963.3 ± 5334.0), followed by the *eglin* family (7050.9 ± 5701.4) and the *hirustasin* superfamily (6398.5 ± 2887.8). The *lumbrokinase* family had the lowest expression level (0.3 ± 0.3), followed by the GGT family (17.5 ± 11.9) and the therostasin family (32.3 ± 53.1). As for *W. pigra*, the *lefaxin* family also had the highest expression level (20,150.9 ± 12,370.5), followed by the *destabilase* family (16,864.1 ± 14,048.7) and the *saratin* superfamily (16,195.5 ± 23,270.1). The *lumbrokinase* family had the lowest expression level (7.2 ± 9.5), followed by the *apyrase* family (25.2 ± 30.0) and the *therostasin* family (27.9 ± 43.5).

**Figure 8:**
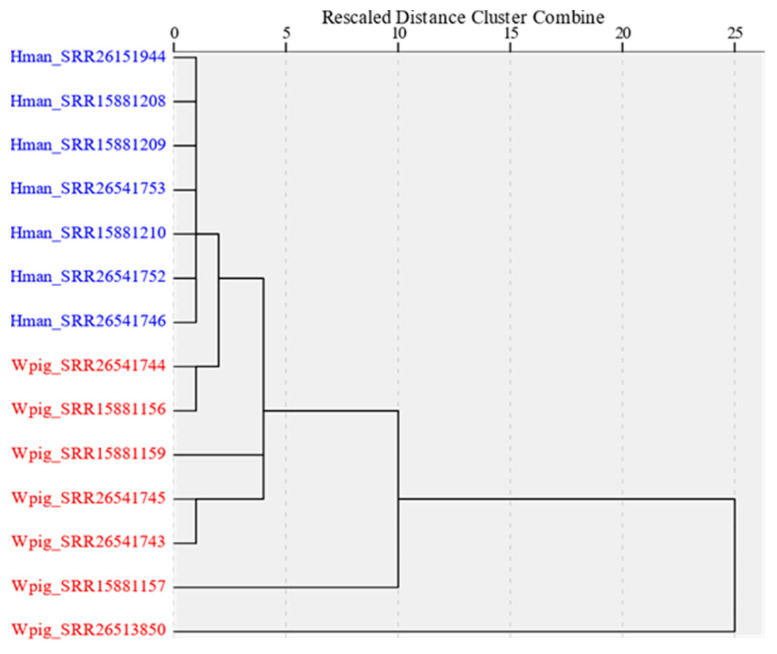
Hierarchical cluster analysis based on the expression of antithrombotic genes two leeches. Blue, seven samples from *H. manillensis*; red, seven samples from *W. pigra*.

The Mann–Whitney U test showed that of the seventeen gene families or superfamilies, nine were significantly deviated in their expression levels (Table 4). In total, five genes, including *hirudin*, *antistasin*, *saratin*, *lumbrokinase*, and *destabilase*, had significantly higher expression levels in *W. pigra* than in *H. manillensis*. In contrast, four gene families, including *eglin*, *bdellin*, *LDTI*, and *apyrase*, had significantly lower expression levels in *W. pigra* than in *H. manillensis*. A hierarchical cluster analysis showed that all of the seven *H. manillensis* samples clustered into a group with small differentiation levels among each other. In contrast, there were much more differentiation levels among the seven *W. pigra* samples, indicating that this species had higher fluctuations in the expression levels of antithrombotic genes (Figure 8).

Hirudin is the first identified and most concerned antithrombotic related gene. Our recent study [27] indicated that, of the five hirudins in *H. manillensis*, *hirudin_Hman1* had the highest anticoagulation activity, followed by *hirudin_Hman5* and *hirudin_Hman2*, while *hirudin_Hman3* and *hirudin_Hman4* had no anticoagulation activity. In this study, expression levels of *hirudins* were rather uneven among the seven *H. manillensis* samples (Supplementary Table S1). For example, *hirudin_Hman1* and *hirudin_Hman2* expressed in only three and two samples, while *hirudin_Hman5* did not express in any of the samples. In contrast, *hirudin_Hman4* and *hirudin_Hman3* expressed in six and two samples. The expression patterns of the hirudins of *W. pigra* (Supplementary Table S2) were more regular than those of *H. manillensis*. The *hirudin_Wpig1*~*hirudin_Wpig6*, whose protein products had no anticoagulation activity, were expressed in most of the seven samples. In contrast, the *hirudin_Wpig7*, whose protein products had anticoagulation activity, were expressed in only two samples (Table 5).

## 4. Discussion

To effectively identify and analyze functional proteins, a high-quality genome is a necessary prerequisite. Combining a third-generation sequencing method and several next-generation sequencing techniques, we obtained a nearly complete chromosome-scale genome of the non-hematophagous leech, *W. pigra*. Similar to the earlier reports, a total of 11 pseudo-chromosomes were found [26]. Nonetheless, the 11 pseudo-chromosomes in our analysis account for 99.77% of the scaffolds’ overall length, which is significantly more than the 94.75% reported in the prior study. According to the BUSCO analyses, 98% of all BUSCOs were successfully captured in our genome. However, upon reexamining the previously released genome with the same settings, it was found that only 92.1% of complete BUSCOs were detected. The Merqury analyses yielded quality value scores of 43.47 in our investigation, which is also greater than the published *W. pigra* genome (35.8) [26]. Furthermore, through sequence assembly based on the survey reads we obtained, a complete circular mitochondrial genome of this species. Therefore, it is highly likely that we have obtained the most complete, whole genomes for *W. pigra* to date based on the aforementioned parameters.

Based on the BRAKER-plus strategy, a total of 24,156 protein-coding genes were predicted from the *W. pigra* genome, including 20 gene families whose protein products were involved in antithrombotic functions. The 79 antithrombotic proteins all shared a sequence similarity of more than 50% with their corresponding archetypal proteins, demonstrating the validity of our identification techniques. Note that the majority of the prior research on antithrombotic proteins or genes has focused on one gene family member at a time. Multigene families actually encoded a large number of proteins. Process-oriented prediction methods frequently omit certain genes, most likely because of the complexity of the antithrombotic genes’ structures. We checked the predicted CDS of *W. pigra* from the previous studies [26,28], and found that only 52 and 32 antithrombotic genes were recovered. The high-quality genome and meticulously tailored analysis in the present study allows a systematic investigation of antithrombotic genes and their corresponding proteins at the gene family level.

Unlike blood-feeding leeches, such as *H. manillensis*, *W. pigra* has completed its habit transition from hematophagous to non-hematophagous [14] and hence was speculated to have reduced antithrombotic genes. Unexpectedly, a total of 79 antithrombotic genes were identified, more than those (72 genes) came from the thorough blood-feeding *H. manillensis*. Similar to *H. manillensis*, these genes can be classified into four categories: (1) coagulation inhibitors, (2) platelet aggregation inhibitors, (3) fibrinolysis enhancers, (4) and tissue penetration enhancers. Of the 21 gene families identified in the *H. manillensis* genome, 20 were found in the *W. pigra* genome, except for the *poecistasin* family. The gene number(s) of twelve gene families were identical between the two species. In six (*hirudin*, *HMEI*, *saratin*, *lumbrokinase*, *destabilase*, and *GGT*) of the remaining nine gene families, *W. pigra* had more gene numbers than *H. manillensis*. An explanation for *W. pigra* having more antithrombotic genes than *H. manillensis*, is that the ancestor of *W. pigra* was a specialized blood feeder, which had more antithrombotic genes than *H. manillensis*, while the evolution time of its habit transition was not long enough to allow it loosing many antithrombotic genes. Phylogenetic analysis showed that *W. pigra* was actually an ingroup of genes of *Hirudo* and had close relationships with *H. nipponia* [54]. Morphological studies also showed that *W. pigra* remained having tooth plates in their jaws [55]. Another explanation is that some of the antithrombotic genes had obtained a function shift, and in order to adapt to the new non-hematophagous habits, these genes exist persistently or even expanded. For example, although there were seven hirudin genes, only one (*hirudin_Wpig7*) corresponding protein had anticoagulation activities. Moreover, four *hirudin* genes (*hirudin_Wpig1*~*hirudin_Wpig4*) were totally identical, indicating they were expanded from a recent gene-duplication event.

Gene expression analyses based on RNA-seq data showed that, for both *H. manillensis* and *W. pigra*, the average TPM of each antithrombotic gene was much higher (18 and 29 times for *H. manillensis* and *W. pigra*, respectively) than the average TPM of the other protein-coding genes, indicating that the antithrombotic genes played an important role in the survival of the two leeches. Interestingly, the average TPM of the antithrombotic genes of *W. pigra* was significantly larger than that of *H. manillensis*. The counterintuitive phenomenon that the non-hematophagous leech had higher overall expression level of antithrombotic genes than the hematophagous leech could also be explained by the two hypotheses mentioned above: (1) the ancestor of *W. pigra* was a specialized blood feeder, which had even higher expression levels of antithrombotic genes than *H. manillensis*, while the evolution time of its habit transition was too short to make a difference; (2) some the antithrombotic genes had obtained function shift, and the new non-hematophagous habits needed higher expression level of these genes. Whether or not these explanations hold true, the large number and high expression levels of the antithrombotic genes suggested that the *W. pigra* had potent antithrombotic capabilities and should be included in the PPRC.

Although the average TPM of the antithrombotic genes of *W. pigra* samples were much higher than that of *H. manillensis*, only five gene families (*hirudin*, *antistasin*, *saratin*, *lumbrokinase*, and *destabilase*) had significantly higher tTPM (total TPM of all gene members within a gene family) values in *W. pigra* than in *H. manillensis*. The most amazing case happened in the *hirudin* family, that is, although it was repeatedly found that *W. pigra* had a much weaker anticoagulation activity than *H. manillensis*, the tTPM of the *hirudin* family of the *W. pigra* was nearly 25 times those of *H. manillensis*. Further analysis found that the six genes (*hirudin_Wpig1*~*hirudin_Wpig6*) whose protein products had no anticoagulation activity constitute most of the total expressions. In contrast, the remaining gene (*hirudin_Wpig7*), whose protein product had anticoagulation activity (Table 2), constitute few (<0.1%) of the total expressions. Considering the expansion of the *hirudin* gene family and the extremely high expression level of *hirudin_Wpig1*~*hirudin_Wpig6*, we suggest that the function of the six genes was no longer for anticoagulation, but for some other functions that were important for *W. pigra* to adapting to its new habits. Similar to *hirudin*, the case of the combination of gene expansion and increased expression level also happened in *saratin*, *lumbrokinase*, and *destabilase*. For example, compared with *H. manillensis* which had two *saratin* genes, the number of *saratin* genes in *W. pigra* expanded into eleven, and their total expression level was nearly eleven times those of *H. manillensis*. The exception case happened in the antistasin family, that is, both *H. manillensis* and *W. pigra* had two antistasin genes, but their expression level in *W. pigra* was about eighteen times of that in *H. manillensis*.

Compared to inorganic or organic small molecules, the structural and functional stability of proteins is relatively weaker. Hence, as a drug with proteins as the main active ingredients, the pharmaceutical efficacy of leech materials will be considerably influenced by different processing methods. Guan et al. [56] reported that the anticoagulating activity of fresh *H. manillensis* was significantly higher than *W. pigra*. After water boiling for one hour, *H. manillensis* thoroughly lost anticoagulation activity; however, the anticoagulation activity of *W. pigra* was almost unaffected. We speculate that the antistasins might be the key components that keep the anticoagulation activated in boiled *W. pigra*, not only because of their higher expression level compared to *H. manillensis*, but also for the extremely rich cysteine in this protein family. Of the ~120 residues in the functional region, 20 cysteines were found. In other words, on average, one cysteine occurs in every six amino acids. Disulfide bonds play a crucial role in proteins as they modulate the stability of proteins and constrain the conformational dynamics of proteins [57]. The high density of disulfide bond formed by the cysteines might have largely increased the stability of antistasins under boiling.

To sum up, the current work offers a high-quality, nearly whole genome of *W. pigra*. A total of twenty antithrombotic gene families related to anticoagulation, antiplatelet aggregation, fibrinolysis, and drug diffusion were found. This is so far the most comprehensive collection of antithrombotic biomacromolecules for a non-hematophagous leech. Moreover, using RNA-seq data, we completed comparative analyses on the relative expression levels of the antithrombotic genes as well as gene families between *W. pigra* and *H. manillensis*. Again, it is the first systematic comparison on the gene expression between a non-hematophagous and a hematophagous leech. Our results will shed light on the research and application of leech derivates for medical and medicinal purposes of thrombosis.

## Figures and Tables

**Table 1:** Number of antithrombotic genes of *Whitmania pigra* (this study) and *Hirudinaria manillensis* (from [27]).

Gene Family	*W. pigra*	*H. manillensis*	Protein Function
*hirudin*	7	5	coagulation inhibitor
*progranulin*	1	1	coagulation inhibitor
*antistasin*	2	2	coagulation inhibitor
*lefaxin*	3	3	coagulation inhibitor
*therostasin*	1	1	coagulation inhibitor
*hirustasin/hirustasin-like*	1/9	1/12	coagulation inhibitor
*guamerin*	1	1	coagulation inhibitor
*piguamerin*	1	1	coagulation inhibitor
*bdellastasin*	1	1	coagulation inhibitor
*poecistasin*	0	2	coagulation inhibitor
*eglin*	2	4	coagulation inhibitor
*bdellin*	1	1	coagulation inhibitor
*LDTI*	1	1	coagulation inhibitor
*HMEI*	20	18	coagulation inhibitor
*saratin*	11	2	platelet aggregation inhibitor
*apyrase*	3	5	platelet aggregation inhibitor
*lumbrokinase*	4	3	platelet aggregation inhibitor
*destabilase*	4	3	fibrinolysis enhancer
*GGT*	2	1	fibrinolysis enhancer
*LCI*	1	1	fibrinolysis enhancer
*hyaluronidase*	3	3	tissue penetration enhancer
total	79	72	—

**Table 2:** Results of online blasting of the hirudins identified from the *W. pigra* genome.

Query Protein	Target Species	Target Protein	Target Accession	Identity	Function
hirudin_Wpig1~4	*W. pigra*	Wpig_V6	USH09350.1	100%	inactive
hirudin_Wpig5	*W. pigra*	Wpig_V2	USH09353.1	98.72%	inactive
hirudin_Wpig6	*W. pigra*	Wpig_V3	USH09356.1	100%	inactive
hirudin_Wpig7	*W. pigra*	Wpig_V1	USH09346.1	100%	active

**Table 3:** Average transcripts per million (TPM) values of the antithrombotic and non-antithrombotic genes of each sample.

Species	Sample	Antithrombotic Genes	Non-Antithrombotic Genes
*H. manillensis*	Hman_SRR26151944	605.8	37.8
	Hman_SRR26541753	794.1	37.3
	Hman_SRR26541752	675.9	37.6
	Hman_SRR26541746	789.4	37.3
	Hman_SRR15881208	742.3	37.5
	Hman_SRR15881209	415.1	38.4
	Hman_SRR15881210	739.5	37.5
	Mean ± SD	680.3 ± 134.2	37.6 ± 0.4
*W. pigra*	Wpig_SRR26513850	2199.1	34.3
	Wpig_SRR26541745	882.7	38.6
	Wpig_SRR26541744	588.2	39.6
	Wpig_SRR26541743	838.1	38.8
	Wpig_SRR15881156	863.8	38.7
	Wpig_SRR15881157	1514.5	36.6
	Wpig_SRR15881159	811.9	38.9
	Mean ± SD	1099.8 ± 562.2	37.9 ± 1.8

**Table 4:** Comparison of the tTPM (total TPM of all gene members within a gene family) of the antithrombotic gene families between the *W. pigra* and *H. manillensis* samples (the bold font indicates the gene families which had significantly higher expression levels).

Gene Family	tTPM (Mean ± SD)		Mann-Whitney U test	
	*H. manillensis*	*W. pigra*	*Z* Value	*p* Value
*hirudin*	523.4 ± 577.8	**12,942.4 ± 12,976.2**	−2.747	0.004
*progranulin*	230.8 ± 119.1	172.9 ± 187.6	−1.214	0.259
*antistasin*	454.0 ± 419.5	**8268.1 ± 7249.5**	−2.364	0.017
*lefaxin*	18,963.3 ± 5334	20,150.9 ± 12,370.5	−0.064	1.000
*therostasin*	32.3 ± 53.1	34.1 ± 46.3	−0.332	0.805
*Hirustasin* #	6398.5 ± 2887.8	6135.1 ± 4635.6	−0.064	1.000
*eglin*	**7050.9 ± 5701.4**	197.2 ± 133.1	−2.619	0.007
*bdellin*	**2611.7 ± 1291.6**	746.1 ± 707.6	−2.747	0.004
*LDTI*	**2312.8 ± 1960.7**	27.9 ± 43.5	−2.364	0.017
*HMEI*	3490.8 ± 2578.1	3618.2 ± 2670.2	−0.064	1.000
*saratin*	1479.3 ± 1886.7	**16,195.5 ± 23,270.1**	−2.619	0.007
*apyrase*	**492.1 ± 597.7**	25.2 ± 30.0	−2.747	0.004
*lumbrokinase*	0.3 ± 0.3	**7.2 ± 9.5**	−2.619	0.007
*destabilase*	3341.9 ± 3137.4	**16,864.1 ± 14,048.7**	−2.875	0.002
*GGT*	17.5 ± 11.9	77.5 ± 86.3	−1.214	0.259
*LCI*	1415.8 ± 1037.7	1333.9 ± 1602.4	−0.192	0.902
*hyaluronidase*	166.6 ± 198.0	84.1 ± 34.9	−0.447	0.710

Note: #, *hirustasin* superfamily was a combination of the gene families including *hirustasin*, *hirustasin-like*, *guamerin*, *piguamerin*, *bdellastasin*, and *poecistasin*.

**Table 5:** Average transcripts per million values of each hirudin gene.

Species	Gene	TPM (Mean ± SD)	Anticoagulation
*H. manillensis*	*hirudin_Hman1*	176.0 ± 300.2	active
	*hirudin_Hman2*	238.8 ± 630.8	active
	*hirudin_Hman3*	3.6 ± 5.6	inactive
	*hirudin_Hman4*	105.0 ± 185.1	inactive
	*hirudin_Hman5*	0.0 ± 0.0	active
*W. pigra* #	*hirudin_Wpig1*	1985.9 ± 2303.0	inactive
	*hirudin_Wpig2*	1985.9 ± 2303.0	inactive
	*hirudin_Wpig3*	1985.9 ± 2303.0	inactive
	*hirudin_Wpig4*	1985.9 ± 2303.0	inactive
	*hirudin_Wpig5*	1892.2 ± 2371.9	inactive
	*hirudin_Wpig6*	3102.4 ± 3593.7	inactive
	*hirudin_Wpig7*	4.0 ± 8.0	active

Note: #, *hirudin_Wpig1~hirudin_Wpig4* were identical, hence their TPMs were calculated as 1/4 of the total TPM of the four genes.

## Data Availability

The sequence data (clean reads) of PacBio, Hi-C, Survey, RNASeq were deposited in GenBank under Project PRJNA1032042 and PRJNA1032729.

## Supplementary Materials

The following supporting information can be downloaded at: https://www.mdpi.com/article/10.3390/genes15020164/s1; supplementary_File_S1_genome.fa: the genome assembly of *W. pigra*; supplementary_File_S2_annotation.gff: the GFF file predicted by the BRAKER-plus approach; supplementary_File_S3_all_CDS.fa: coding sequences of all predicted protein-coding genes; supplementary_File_S4_antithrombotic_CDS.fa: coding sequences of the 79 antithrombotic genes; supplementary_Figure_S1_to_S11.docx: sequence alignments of the antithrombotic protein families not shown in the main text; supplementary_Table_S1_and_S2.xlsx: TPM values of the *H. manillensis* and *W. pigra* samples.
