# Supplementary material for: A Chromosome-Level Genome Assembly of the Non-Hematophagous Leech Whitmania pigra (Whitman 1884): Identification and Expression Analysis of Antithrombotic Genes
Source: Genes (Basel). 2024 Jan 26;15(2):164. doi: 10.3390/genes15020164 (PMC10887747; doi:10.3390/genes15020164)
Supplement: Supplementary file 1 [file genes-15-00164-s001.zip › supplementary_Figure_S1_to_S11.pdf]

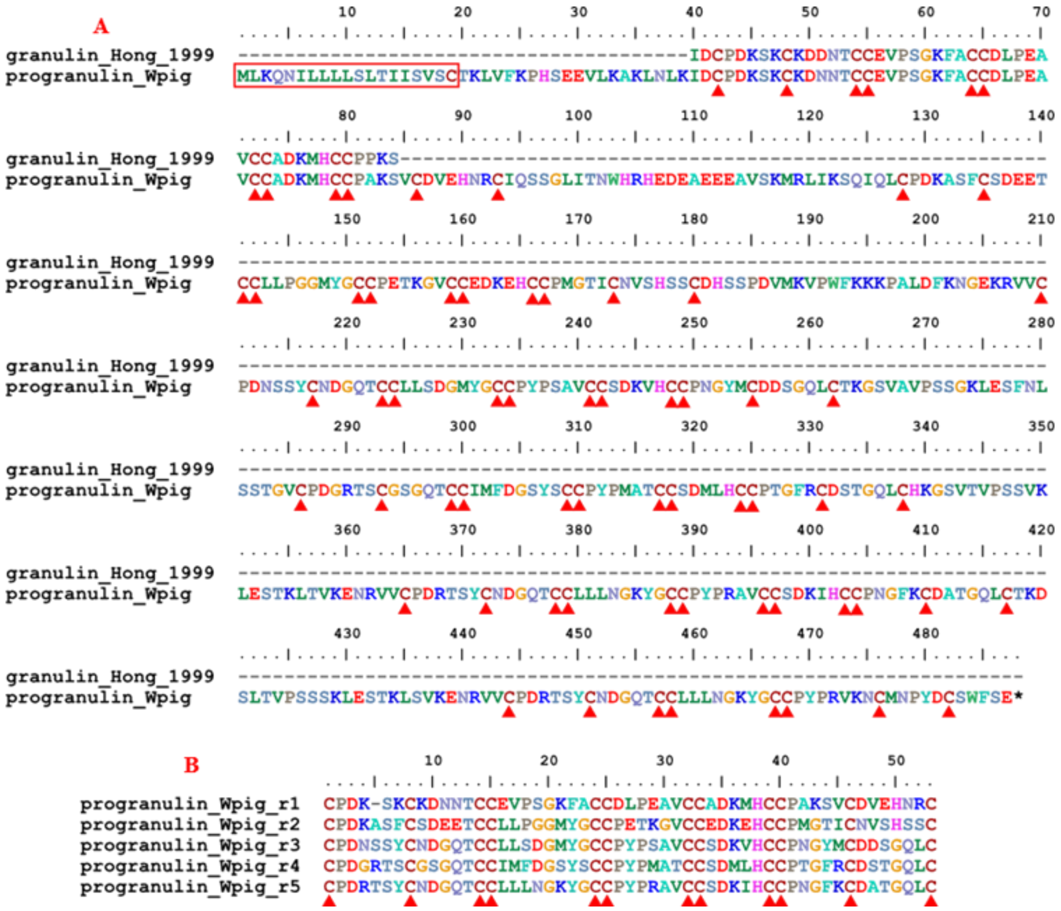

Figure S1. Sequence alignment of granulin and progranulin. (A) Alignment of the archetypal granulin from *Hirudo nipponia* and the progranulin identified from the *W. pigra* genome; (B) Alignment of the tandem repeats within the progranulin. Note: the archetypal granulin is a short partial segment that is located in the N-terminus of the protein.

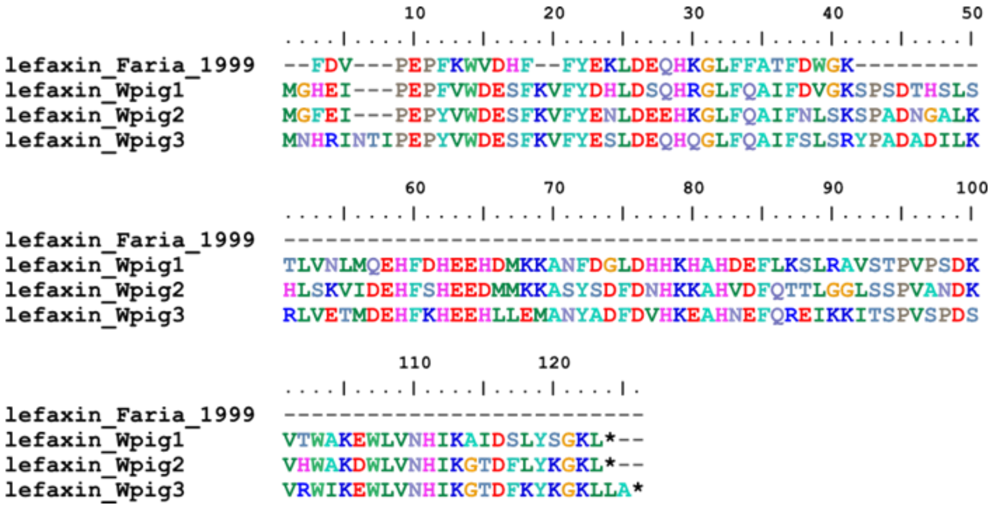

Figure S2. Sequence alignment of lefaxins. Alignment of the archetypal lefaxin first discovered from *Haementeria depressa* and the three lefaxins identified from the *W. pigra* genome.

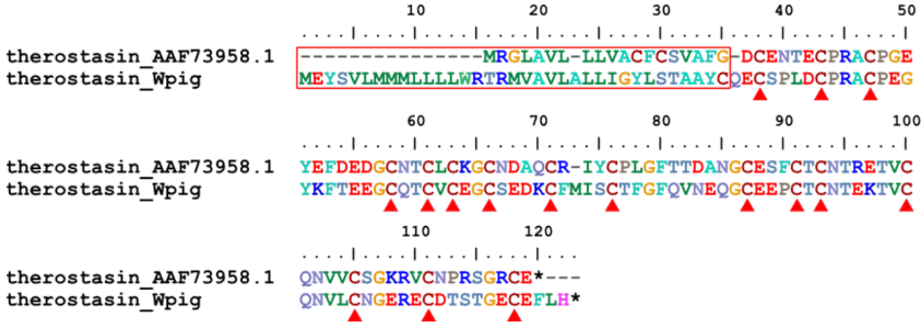

Figure S3. Sequence alignment therostasins. Alignment of the archetypal therostasin from *Theromyzon tessulatum* and the therostasin identified from the *W. pigra* genome.

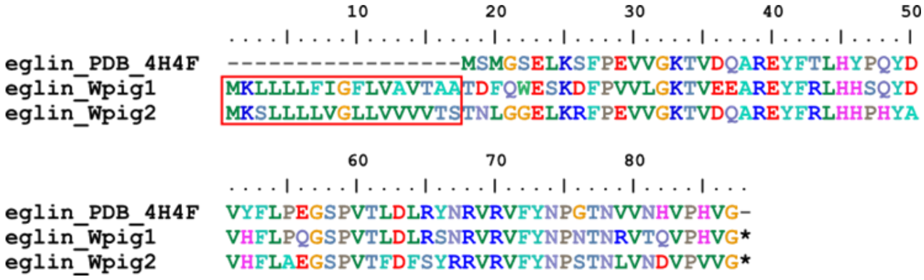

Figure S4. Sequence alignment of eglins. Alignment of the archetypal eglin first discovered from *H. medicinalis* and two eglins identified from the *W. pigra* genome.

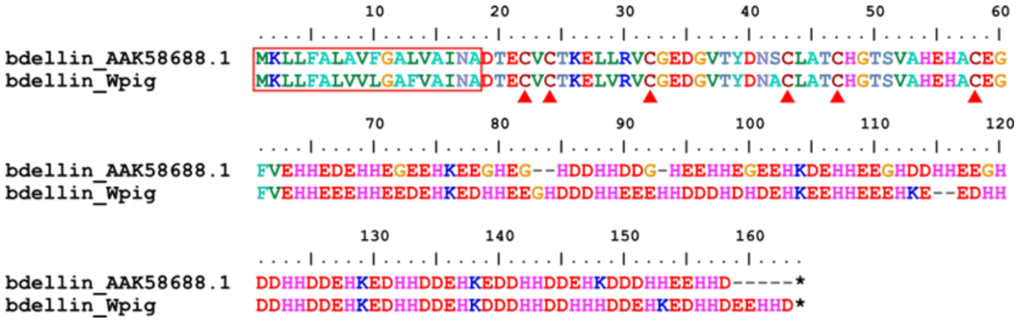

Figure S5. Sequence alignment of bdellins. Alignment of the archetypal bdellin from *H. medicinalis* and the bdellin from the *W. pigra* genome.

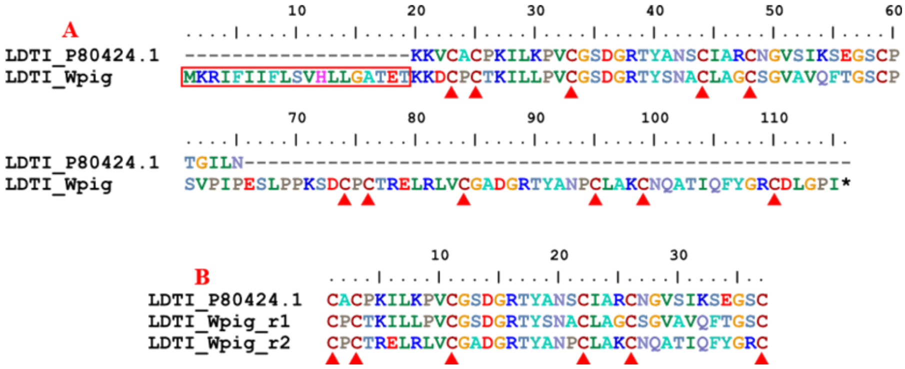

Figure S6. Sequence alignment of LDTIs. (A) Alignment of the LDTI from *H. medicinalis* and that from the *W. pigra* genome; (B) Alignment of the internal tandem repeats of the *W. pigra* LDTI.

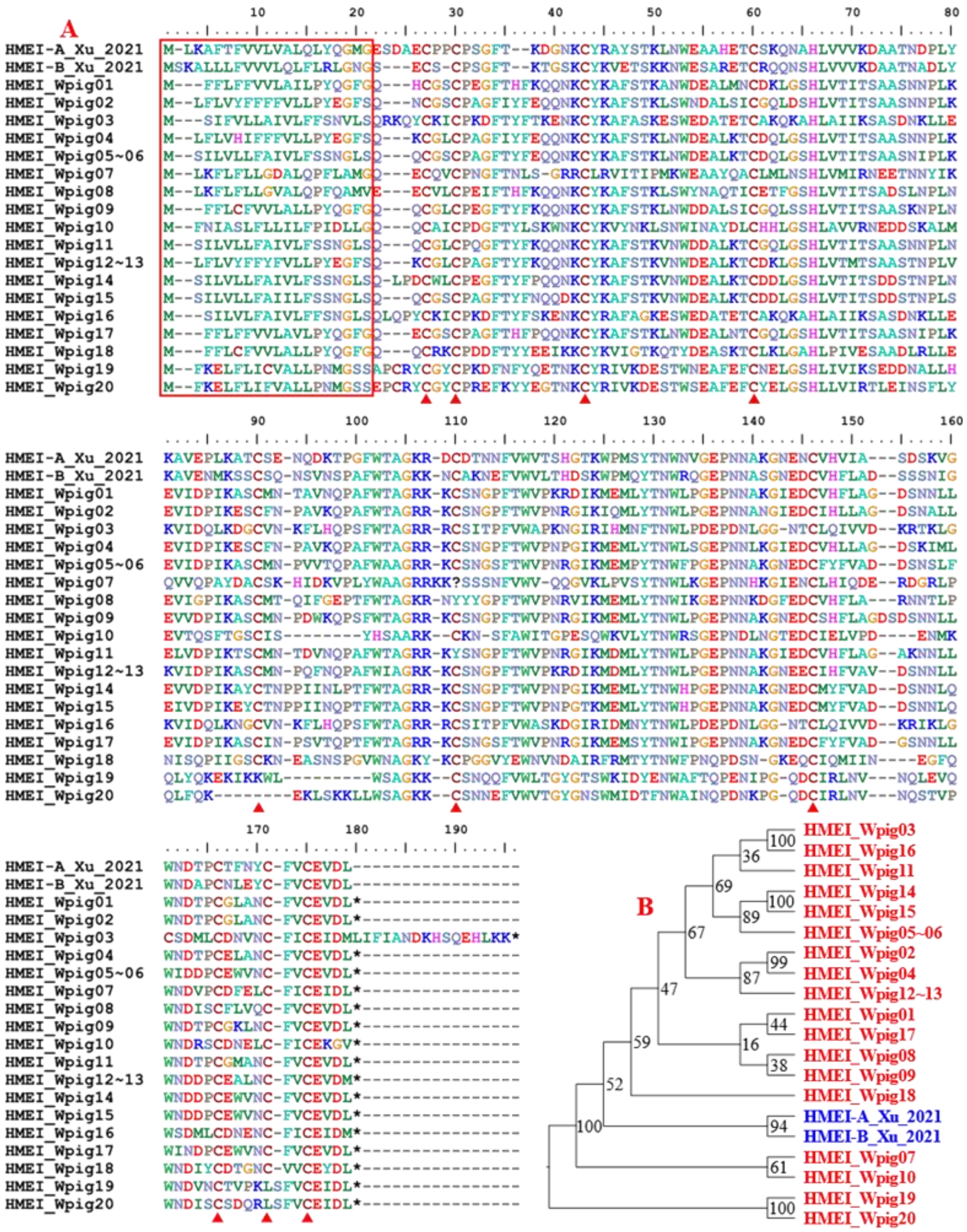

Figure S7. Sequence alignment and phylogenetic relationship of HMEIs. (A) Alignment of the HMEI-A and HMEI-B from *H. manillensis* and the HMEIs identified from the *W. pigra* genome; (B) phylogenetic relationships of the HMEIs.

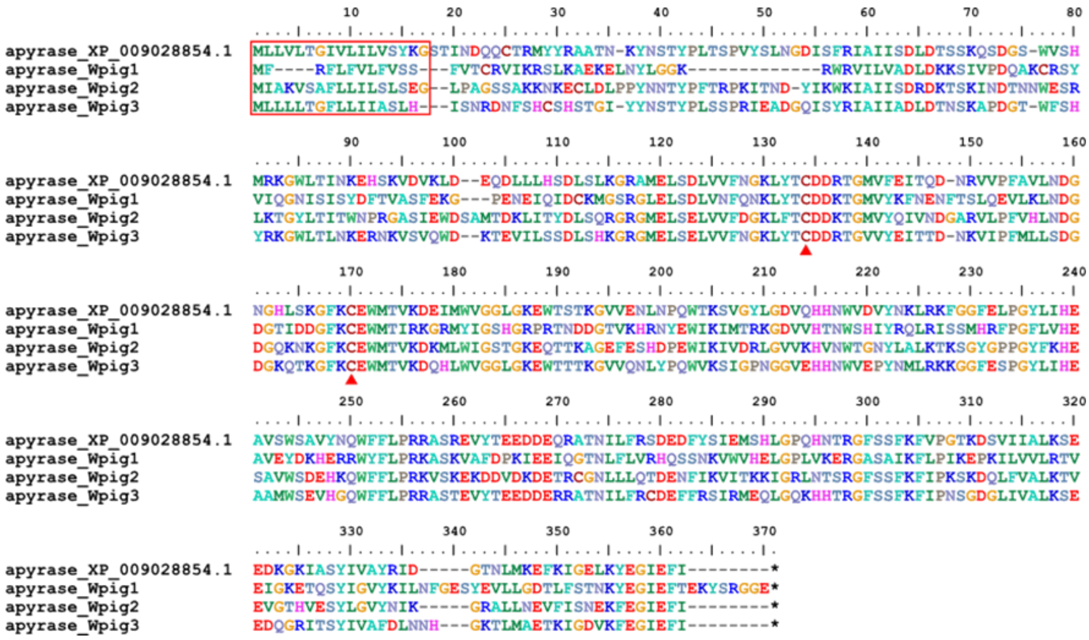

Figure S8. Sequence alignment of apyrases. Alignment of the apyrase from *Helobdella robusta* and those from the *W. pigra* genome.

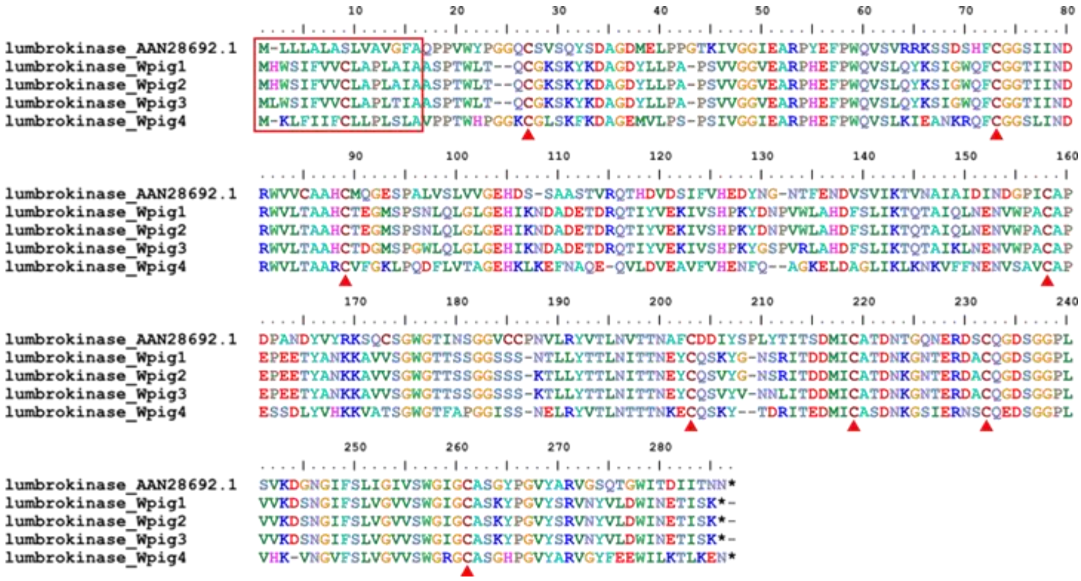

Figure S9. Sequence alignment of lumbrokinases. Alignment of the lumbrokinase from *Lumbricus rubellus* and those identified from the *W. pigra* genome.

```
                           10        20        30        40        50        60        70        80
                   ....|....|....|....|....|....|....|....|....|....|....|....|....|....|....|....|
GGT_Friedrich_1998 MAAKGLSANQ-IITASACILICTSMALGVGLTYGLRGNKASDTPSPFTPSAYKLFSLYAVATETVACSKIGKDIIK----
GGT_Wpig1          MAAKGLSTNQWIIIASACILICTAVALAVGLTYGLRDNKSSDTPTPFTPSAYKLFSLYAVATDTVACSKIGKDIIKNNGT
GGT_Wpig2          MAAKGSSPNRWIIIASACFLICAAMALGDGLTYGRRYNKSSDTPAPFTPSAYKLFSHYAVATETVVCSKIGKDIIKKNGT

                           90       100       110       120       130       140       150       160
                   ....|....|....|....|....|....|....|....|....|....|....|....|....|....|....|....|
GGT_Friedrich_1998 --------------------------------------------------------------------------------
GGT_Wpig1          VVDSAIASMLCVGVISAHSNGIGGGFVSIIYAKPNNDTKKELVTLIARERAPQKAYQKMFASKSSLYGGLAAGIPGEIKG
GGT_Wpig2          VVDSAIASMLCIGVISAHSNGIGGGFVSIIYAKPKHGSKKELVTLIARERAPQKAHQKMFANKSSLIGGLASGIPGEIKG

                          170       180       190       200       210       220       230       240
                   ....|....|....|....|....|....|....|....|....|....|....|....|....|....|....|....|
GGT_Friedrich_1998 --------------------------------------------------------------------------------
GGT_Wpig1          YYEAWKRFGRLPWKELFQPTIDLLEKGYVIEKVLSSTLDTKSLAQLVLNSSLVKSSLLRETYYPNGQPLKAGDTLKDQRL
GGT_Wpig2          YYEAWKRFGRLPWKELFQPTIDLLENGYVIEKVLSSTLDTKSLAQLVLNSSLVKSSLLRDTYYPNGQPLKAGDTLKDQRL

                          250       260       270       280       290       300       310       320
                   ....|....|....|....|....|....|....|....|....|....|....|....|....|....|....|....|
GGT_Friedrich_1998 --------------------------------------------------------------------------------
GGT_Wpig1          ADTFKKIAQNPESFYNGDLAKTIAQEIQNEGGIITEEDLGNYTVEWKKPIEVFLNGSNTRLYSVRPPASGAVLGLMLNIL
GGT_Wpig2          ADTFKKIV*NPESFYNGDLAKTIAREIQNEGGIITEEDLANYKVEWNKPIEVFLNGKKTRLYSVGPPASGAVLGLIMNIL

                          330       340       350       360       370       380       390       400
                   ....|....|....|....|....|....|....|....|....|....|....|....|....|....|....|....|
GGT_Friedrich_1998 --------------------------------------------------------------------------------
GGT_Wpig1          KNYNFTPSSVNDDSKVLTFHRIVEAFRFAYAGRLELGDENFTNVTDLISNMTSEKFAKELWQKIDDSKTQQPDDYRANYA
GGT_Wpig2          KHYNFKPSSVNDDSKVLTFHRILEAFRFAYAGRLELGDENVANVTDLISNMTSEKFAKELWQQIDDSKTQQPDDYRANYA

                          410       420       430       440       450       460       470       480
                   ....|....|....|....|....|....|....|....|....|....|....|....|....|....|....|....|
GGT_Friedrich_1998 --------------------------------------------------------------------------------
GGT_Wpig1          PWADHGTAHLSILAPDGSAVAMTSTINTKFGSLVKGPITGIIYNNQMDDFSSPDQNNFYGYTPSPRNFIVPGKRPMSSMT
GGT_Wpig2          PWADHGTAHVSILAPDGSAVAMTSTINTEFGSLVKGPITGIIYNNQMDDFSSPGQSNFFGYTPSPRNFIVPGKRPMSSMT

                          490       500       510       520       530       540       550       560
                   ....|....|....|....|....|....|....|....|....|....|....|....|....|....|....|....|
GGT_Friedrich_1998 --------------------------------------------------------------------------------
GGT_Wpig1          PTLILDDQDTVRFVAGASGGSRIPTTTAWVIINSLWFNLTLGQAVDFPRLHDQLLPNITYYESRSEPAVIKGLVKKGHTV
GGT_Wpig2          PTLILDDQDTVRFVAGASGGSRIPTTTAWVIINSLWFNLTLGQAVDFPRLHDQLLPNITYYESRSEPAVIKGLVKKGHTV

                          570       580       590       600
                   ....|....|....|....|....|....|....|....|..
GGT_Friedrich_1998 ------------------------------------------
GGT_Wpig1          LADDYLIGVSQSIRNWCRKPLEESCIEAVSDTRKFGVPYGF*
GGT_Wpig2          LADDYLIGVSQSIRNWCRKPLEKGCIEAVSDTRKFGVPYGL*
```

Figure S10. Sequence alignment of GGTs. Alignment of the archetypal GGT from *H. medicinalis* and those from *W. pigra* (the red dot shows the site of early termination; the red star shows the catalytic threonine).

```
                         10        20        30        40        50
                    ....|....|....|....|....|....|....|....|....|....|
LCI_Reverter_2000   ---------------GSHTPDESFLCYQPDQVCCFICRGAAPLPSEGECN
LCI_Wpig            MKFLIVLLCFLQLVISSYTPDESFLCYQSDQVCCFICREAVPLESEGECS

                         60        70        80
                    ....|....|....|....|....|...
LCI_Reverter_2000   PHPTAPWCREGAVEWVPYSTGQCRTTCIPYVE-
LCI_Wpig            PSPIRPWCQEGAVEWIPYSTGQCRTTCIPYSQ*
```

Figure S11. Sequence alignment of LCIs. Alignment of the archetypal LCI (from *H. medicinalis*) and the LCI identified from the *W. pigra* genome.
